# Free convective trickling over a porous medium of fractional nanofluid with MHD and heat source/sink

**DOI:** 10.1038/s41598-022-25063-y

**Published:** 2022-12-01

**Authors:** Yuanjian Lin, Sadique Rehman, Nevzat Akkurt, Tim Shedd, Muhammad Kamran, Muhammad Imran Qureshi, Thongchai Botmart, Abdulaziz N. Alharbi, Aamir Farooq, Ilyas Khan

**Affiliations:** 1 Nanchang Normal College of Applied Technology, Nanchang, 330108 China; 2 Nanchang Institute of Science and Technology, Nanchang, 330108 China; 3 Division of Mathematical and Physical Sciences, Kanazawa University, Kakuma, Kanazawa 920-1192 Japan; 4 Rare Earth Elements Applications and Research Center, Munzur University, 62000 Tunceli, Turkey; 5 Engineering Technologist, Office of the CTO, Dell Technologies, Austin, TX USA; 6 Department of Mathematics, COMSATS University Islamabad, Wah Campus, Wah, 47040 Pakistan; 7 Department of Mathematics, COMSATS University Islamabad, Vehari Campus, Vehari, 61100 Pakistan; 8 Department of Mathematics, Faculty of Science, Khon Kaen University, Khon Kaen, 40002 Thailand; 9 Department of Physics, College of Science, Taif University, P. O. Pox 11099, Taif, 21944 Saudi Arabia; 10 Department of Mathematics, Abbottabad University of Science and Technology, Abbottabad, Pakistan; 11 Department of Mathematics, College of Science Al-Zulfi, Majmaah University, Al-Majmaah, 11952 Saudi Arabia

**Keywords:** Engineering, Mathematics and computing, Nanoscience and technology, Physics, Applied physics

## Abstract

Nanofluids are considered as smart fluids that can improve heat and mass transfer and have numerous applications in industry and engineering fields such as electronics, manufacturing, and biomedicine. For this reason, blood-based nanofluids with carbon nanotubes (CNTs) as nanoparticles in the presence of a magnetic field are discussed. The nanofluid traverses the porous medium. The nanofluids move on a vertical plate that can be moved. The free convection heat transfer mode is considered when the heat source and heat fluxes are constant. Convective flows are often used in engineering processes, especially in heat removal, such as geothermal and petroleum extraction, building construction, and so on. Heat transfer is used in chemical processing, power generation, automobile manufacturing, air conditioning, refrigeration, and computer technology, among others. Heat transfer fluids such as water, methanol, air and glycerine are used as heat exchange media because these fluids have low thermal conductivity compared to other metals. We have studied the effects of MHD on the heat and velocity of nanofluids keeping efficiency in mind. Laplace transform is used to solve the mathematical model. The velocity and temperature profiles of MHD flow with free convection of nanofluids were described using Nusselt number and skin friction coefficient. An accurate solution is obtained for both the velocity and temperature profiles. The graph shows the effects of the different parameters on the velocity and temperature profiles. The temperature profile improved with increasing estimates of the fraction parameter and the volume friction parameter. The velocity of the nanofluid is also a de-escalating function with the increasing values of the magnetic parameter and the porosity parameter. The thickness of the thermal boundary layer decreases with increasing values of the fractional parameter.

## Introduction

Nowadays, most researchers and scientists pay great attention to those methods and techniques that are useful for improving heat transfer in various heat exchanger processes. To meet these requirements, researchers have developed a new type of fluid called a nanofluid. A nanofluid is a fluid that contains nanoparticles, which are nanometer-sized particles. Metals, their oxides, carbides and carbon nanotubes are the most commonly used nanoparticles in nanofluids. Nanofluids are helpful and have a wide range of applications, including microelectronics, fuel cells, pharmaceutical processes, cross-race machines, temperature controls, heating systems, exhaust gasses from smokestacks, heat dissipation, and so on. Due to the importance of nanofluids, numerous experimental and theoretical observations are being carried out by many researchers. In a detailed study, Kakac et al.^1^ investigated how nanofluids increase the thermal conductivity of a base fluid. Due to the high predictability of nanofluids, the problems identical to decay, clumping of new charges, and sedimentation do not occur^2^. In recent years, researchers have focused on the thermal perspectives of nanofluid because it is practical and has more applications in heat transfer and cooling. Natural convection is the general mode of heat movement. The phenomenon of natural convection allows heat to flow with external aids such as suction devices, fans, and pumps, etc., and these flows are created by changing the density of fluids. It has been observed that as the temperature changes, the density decreases, but the volume increases, so that the heated layer is loses its thickness and rises. In nature, free convection currents usually occur, caused by differences in concentration and density. The most important works and reviews by researchers can be such as Ghosh and Beg^3^ studied the effects of local thermal non-equilibrium (LTNE) on free convection in a uniformly curved, non-Darcian permeable annulus traversed by nanofluid. Fetecau et al.^4^ used an isothermal vertical plate to study a fractional nanofluid combining the effects of thermal radiation and natural convection, and found the solution of the temperature and dimensionless velocity using the Laplace transform and Caputo-Fabrizio time derivative. Toki and Tokis^5^ studied the free convection flow considering the time-dependent heating over a porous medium and used the Laplace transform to find an exact solution. Hussanan et al.^6^ studied mass and heat transfer using a vertical plate and a Newtonian heater and presented an accurate temperature and velocity analysis that satisfied the boundary conditions. Turkyilmazoglu and Pop^7^ studied a nanofluid over a vertical flat (infinite) surface in natural convection flow with radiation effect. Pramanik^8^ found a result for a Casson fluid flowing through a stretching surface exponentially porous under the influence of thermal radiation. Turkilmazgolu^9^ studied the effect of heat transfer and unsteady flow of a nanofluid through a moving vertical plate. Ge-JiLe et al.^10^ studied the radiated MHD flow of iron-containing nanoparticles with Brownian motion and thermophoresis through a cone. Kavya et al.^11^ revealed a hybrid nanofluid with MHD and heat extraction/injection through a shrinking/stretching cylinder with a suspension of MoS4 and copper nanoparticles. The study of a hybrid nanofluid composed of a Newtonian and a non-Newtonian fluid flowing over a stretching sheet was reported by^12–17^.

The magnetic field affects both man-made and natural currents. The magnetic field plays a major role in the pumping, stirring, and levitation of liquid metals and in the generation of electricity in industry. Molten metals are found in the Earth's core, creating a magnetic field known as the geomagnetic field. Sunspots and solar flares form the solar magnetic field. Due to practical applications, the study of MHD with heat transfer is of particular importance, as evidenced by the buoyancy-induced effect in quasi-solid bodies, water bodies, and the atmosphere, e.g., the Earth. Khan et al.^15^ studied the unsteady MHD flow with free convection in a porous medium with heat diffusion and a sloped wall. Khan et al.^16^ also considered a porous medium with Newtonian heating and a Casson type sodium alginate based nanofluid and analyzed the unsteady MHD flow. Yigra et al.^17^ dealt with mass transfer and convective heat in a nanofluid in applied magnetic field, flow past a permeable medium on a stretching sheet, chemical reaction, viscous dissipation and Soret effect. Gaffar et al.^18^ studied MHD (free convection) flow with ohmic dissipation of Eyring-Powell fluid and Hall/Islip flows in a porous medium on a vertical surface. Mahmoudi et al.^19^ obtained a result to improve the heat transfer and entropy generation in a flow with natural convection using a copper–water nanofluid and a two-dimensional trapezoidal enclosure with a continuous magnetic field. Khan et al.^20^ considered a non-compressible fluid (viscous) and worked on the results of MHD flow with free convection in a permeable medium located near an oscillating plate. Jha et al.^21^ used a vertical annular microchannel in which a magnetic field is present and discussed free convection flow. Sheikholeslami et al.^22^ inquired about the flow behavior using a constant heat source and a porous medium and obtained results for a nanofluid by increasing the buoyancy forces to enhance the heat transfer. In the applied magnetic field, Fetecau et al.^23^ studied the natural convection flow with radiation effects. Zeeshan et al.^24^ studied the spontaneous convection flow through porous media under the influence of MHD and provided pictorial and mathematical results. Ashorynejad et al.^25^ studied hybrid nanofluid as natural convection flow in an open cavity under the influence of MHD. Turkilmazgolu^26^ studied the heat transfer and mass properties of electrically conducting fluids over a apartment plate (infinite and vertical) and represented them numerically. Sheikholeslami et al.^27^ studied the effects of MHD on natural convection in a 2D horizontal annulus for an Al2O3-water nanofluid. Azhar et al.^28^ discussed a fractional nanofluid as a free convection system with a constant heat flux and a heat source flowing over an endless vertical plate, focusing on the graphical and analytical results.

Wang et al.^29^ studied the heat and mass transfer of a general MHD-Oldroyd-B bio-nanofluid in a permeable medium with increasing conditions in comparison. Heat transport by free convection is an important branch of fluid dynamics that has been matured for applications such as geothermal, geo- and astrophysics, paramedical sciences, and oil reservoirs, etc. Ramudu et al.^30^ have studied the influence of Soret and Dufour on Casson MHD fluid flow on an extended surface. The solution of the model is obtained by the Runge–Kutta method (along shooting). Farooq et al.^31^ presented the free convective flow of an oscillating Maxwell nanofluid with heat and mass transport. The velocity is a decreasing function of the volume fraction, while the temperature profile grows with varying estimates of the volume fraction parameter. Tang et al.^32^ reported the comparative approach of naturally convective flow of a fractional Maxwell fluid with radiation and uniform heat flux. The well-known integral transform (Laplace transform) is used to solve the fractional Caputo and Caputo-Fabrizio model. The phenomenon of heat absorption/consumption has numerous applications in engineering such as reinforcement of thrust bearings, cooling of metal sheets, recovery of unpolished oil and in medicine etc. Anantha Kumar et al.^33^ studied the first and second order slips in micropolar fluid flow over a convective surface with MHD and varying heat absorption/consumption. The velocity of the fluid increases as the second order slip is estimated, while the temperature decreases against the second order slip. Anantha Kumar et al.^34^ studied the MHD Cattaneo-Christov flow with variable heat source/sink over a cone and wedge. The study of non-Newtonian MHD fluid flow with heat absorption/consumption along different geometries was analyzed by^35–37,41–46^. Anantha Kumar et al.^38^ studied the MHD fluid Williamson with variable heat source/sink and chemical reaction on a curved/apartment surface. Also Anantha Kumar et al.^39,40^ presented the influence of free convection and nonlinear radiation of a micropolar MHD fluid near stagnation with convective surface.

From the literature review, no work has been done on convective heat transport of nanofluids along a porous medium under the effect of magnetism. Such geometries have many applications in science and technology, such as power generation, conductive plates, automobiles, refrigeration, power generation, etc. Blood is used as the base fluid for the suspension of CNTs. Carbon nanotubes (CNTs) as nanoparticles have great applications in the field of nanotechnology due to their unique electrical shape and mechanical properties. Applications of CNTs also include energy storage, conductive films, advanced electrodes, catalyst supports, coatings, biomedical and sensing applications, wearable electronics, solar and structural materials. CNTs have higher conductivity, which they use to build a network of conducting tubes. To identify the memory effect of nanofluids, the fractional derivative (Caputo-Fabrizio model) is solved exactly using Laplace technique (LT). Finally, various physical parameters are explained physically and graphically. The skin fraction and the Nusslet value are also obtained to determine the rate of heat transport and the drag forces of the nanofluid. Zakian's algorithm is used to simulate graphs and tables^41^.

The research questions are as follows, which is helpful in understanding the novelty and key research findings;

- How do the SWCNTs and MWCNTs nanoparticles affect the flow of a viscous nanofluid with free convection?
- How does the Lorentz force affect the velocity of the nanofluid when magnetic parameters are used?
- How can the exact solution of the fractional model be determined and the memory effect on the nanofluid be established?
- How does the porosity parameter behave on the velocity of the nanofluid?
- How does the fractional parameter affect the thickness of the thermal boundary layer?

## Mathematical statement of the problem

The equations for the free convection flow of an incompressible MHD fluid and the heat transfer in the presence of a heat source/sink at an infinite vertical plate in a porous medium subject to the Boussinesq approximation are as follows,

1 $$\nabla .{\mathbf{V}} = 0,$$

2 $$\rho_{nf} \left[ {\frac{{\partial {\mathbf{V}}}}{\partial t} + \left( {{\mathbf{V}}.\nabla } \right){\mathbf{V}}} \right] = \mu_{nf} \nabla^{2} {\mathbf{V}} + {\mathbf{J}} \times {\mathbf{B}} + {\mathbf{r}} + \rho_{nf} g\beta_{nf} \left( {T - T_{\infty } } \right),$$

3 $$\left( {\rho C_{p} } \right)_{nf} \left[ {\frac{\partial T}{{\partial t}} + \left( {{\mathbf{V}}.\nabla } \right)T} \right] = k_{nf} \nabla^{2} T + {{\varvec{\uptau}}}{\mathbf{.L}} - Q^{ * } \left( {T - T_{\infty } } \right).$$

where, $${\mathbf{r}}$$ denotes the Darcy’s resistance, $${\mathbf{J}}$$ is the current density, $${\mathbf{B}}$$ demonstrates the total magnetic field, $${\mathbf{V}}$$ denotes the velocity vector i-e $${\mathbf{V}} = \left[ {W\left( {Y,\tilde{t}} \right),0,0} \right],$$$${{\varvec{\uptau}}}{\mathbf{.L}}$$ represents the term viscous dissipation, $${\mathbf{L}} = {\text{grad}}{\mathbf{V}}$$, $${{\varvec{\uptau}}}$$ denotes the Cauchy stress tensor i-e $${{\varvec{\uptau}}} = - {\rm P}{\rm I} + S,$$$${\rm P}$$ is the pressure, $${\rm I}$$ represents the unit tensor, $$S$$ expressed the extra stress tensor, $$\rho_{nf} ,\;\mu_{nf} ,\;\beta_{nf} ,\;\left( {C_{p} } \right)_{nf} ,\;k_{nf}$$ are respectively density, absolute viscosity, thermal expansion coefficient of nanofluid, specific heat and nanofluid’s thermal conductivity, $$g$$ is the gravitational acceleration and $$Q^{ * }$$ denotes the coefficient of heat source/sink.

**Figure 1:**
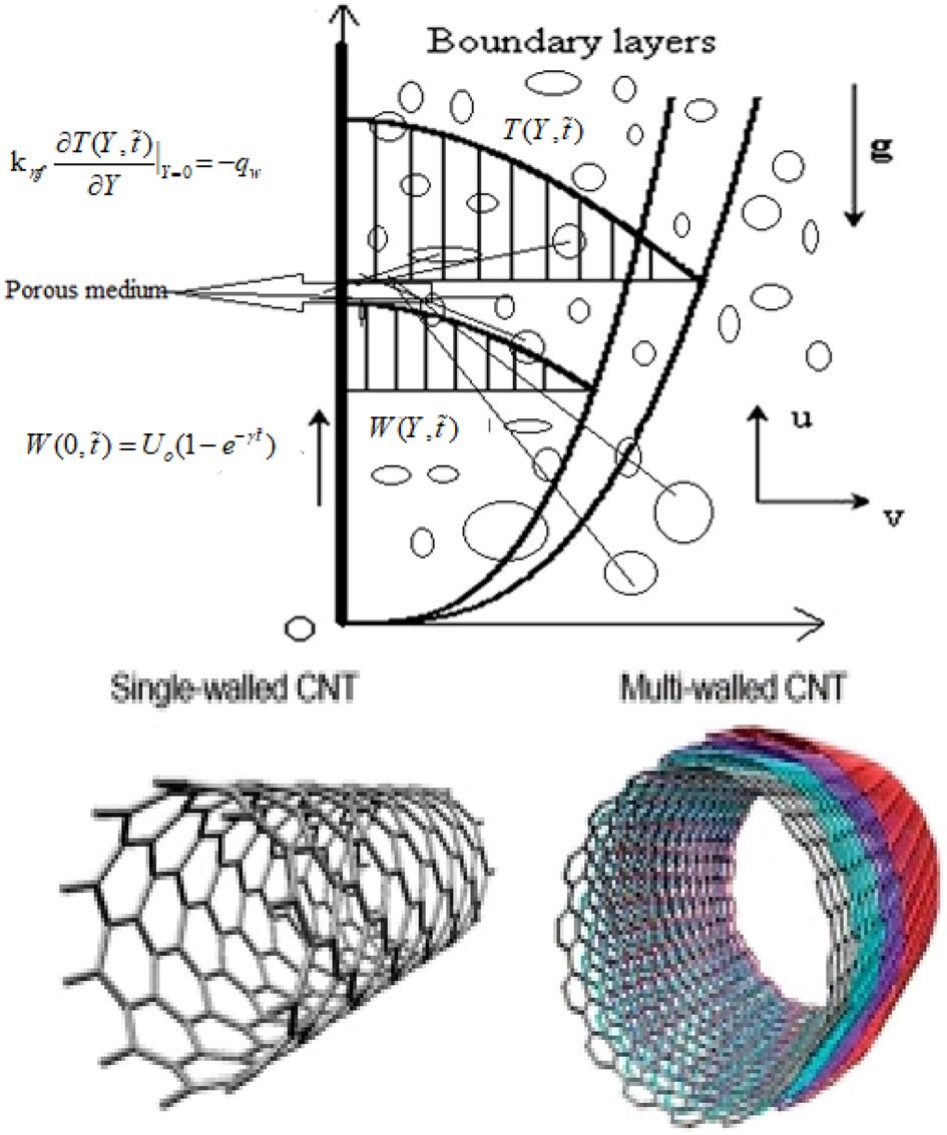
Geometry of the flow problem.

Consider the natural convection flow of electrically conducting and incompressible nano-fluids. The flow medium is an infinite vertical plate. The magnetic field strength B_o acts uniformly and perpendicularly on the plate. At a given time, both the plate and the fluid are in a stationary position with ambient temperature. When the timecomes, the plate starts to move with velocity ${U}_{o}(1-{e}^{-\gamma t})$, provided that no heat enters or leaves the system. Here shows the amplitude of the motion and denotes the dimensional constant. The non-Darcian modal with porous medium is considered. In the energy equation, viscous dissipation is not included because of its small size. The geometry of the flow problem is shown in Fig. 1. In addition, the assumptions made to idealize the above model are examined as follows:

The nanofluid consists of the base fluid blood and nanoparticles called SWCNTs and MWCNTs.

- The thermal equilibrium is balanced between the base fluid and the nanoparticles.
- The temperature buoyancy force in the momentum equation is a function of density.
- The viscous dissipation is ignored in the energy equation.
- The resulting magnetic field due to the nanofluid flow is neglected compared to the imposed magnetic field.
- The influence of the polarization of the nanofluid is ignored, so no external electric field is applied.

However, one-dimensional and unidirectional flow is studied, and the vertical plate is assumed to be infinite in length, so temperature and velocity are only a function of and Darcy's law for viscous fluids is represented as follows

4 $${\mathbf{r}} = - \frac{{\mu_{nf} }}{K}W\left( {\tilde{t},Y} \right).$$

5 $${\text{div}}{\mathbf{B}} = 0,{\text{ curl}}{\mathbf{B}} = \mu_{m} {\mathbf{J}},{\text{ Curl}}{\mathbf{E}} = - \frac{{\partial {\mathbf{B}}}}{\partial t},$$

and utilization of Ohm’s law that lead to,

6 $${\mathbf{J \times B}} = - \left( {\sigma_{nf} B_{0}^{2} W,0,0} \right).$$

The suspension of nanoparticles in a fluid cannot be left uncontrolled; it must be controlled or tightened. Fluid movement and temperature are components of and because they are interdependent. Blood (as the base fluid) plus nanoparticles SWCNTs and MWCNTs form the nanofluid. Table 1 lists the physical and thermal properties of the particles.

**Table 1:** Shows the thermo-physical characteristics of human blood and nanoparticles.

Thermo-Physical properties	Human blood (base fluid)	SWCNTs (nanoparticles)	MWCNTs (nanoparticles)
$\rho \left( {kg/m^{3} } \right)$	1053	2600	1600
$C_{p} \left( {J/kgK} \right)$	3594	425	796
$k\left( {W/mK} \right)$	0.492	6600	3000
$\sigma \left( {S/m} \right)$	0.8	$10^{6} - 10^{7}$	$1.9 \times 10^{ - 4}$
$\beta \times 10^{5} \left( {1/K} \right)$	0.18	$27$	$44$

In response to Eqs. (4)–(6) and all the assumptions mentioned, Eqs. (2) and (3) for nanofluids can be considered as follows^28^;

7 $$\overset{\lower0.5em\hbox{$\smash{\scriptscriptstyle\frown}$}}{\rho }_{nf} \frac{{\partial W(Y,\tilde{t})}}{{\partial \tilde{t}}} = \overset{\lower0.5em\hbox{$\smash{\scriptscriptstyle\frown}$}}{\mu }_{nf} \frac{{\partial^{2} W(Y,\tilde{t})}}{{\partial Y^{2} }} - W(Y,\tilde{t})\overset{\lower0.5em\hbox{$\smash{\scriptscriptstyle\frown}$}}{\sigma }_{nf} \mathop B\nolimits_{o}^{2} - W(Y,\tilde{t})\frac{{\overset{\lower0.5em\hbox{$\smash{\scriptscriptstyle\frown}$}}{\mu }_{nf} }}{K} + g\left( {\overset{\lower0.5em\hbox{$\smash{\scriptscriptstyle\frown}$}}{\rho } \overset{\lower0.5em\hbox{$\smash{\scriptscriptstyle\frown}$}}{\beta } } \right)_{nf} \left( {T(Y,\tilde{t}) - T_{\infty } } \right),$$

8 $$\left( {\overset{\lower0.5em\hbox{$\smash{\scriptscriptstyle\frown}$}}{\rho } C_{p} } \right)_{nf} \frac{{\partial T(Y,\tilde{t})}}{{\partial \tilde{t}}} = \overset{\lower0.5em\hbox{$\smash{\scriptscriptstyle\frown}$}}{k}_{nf} \frac{{\partial^{2} T(Y,\tilde{t})}}{{\partial Y^{2} }} - Q^{ * } (T(Y,\tilde{t}) - T_{\infty } ),Y,\tilde{t} > 0.$$

Here $$\overset{\lower0.5em\hbox{$\smash{\scriptscriptstyle\frown}$}}{\sigma }_{nf}$$, $$K$$ are respectively the electrical conductivity of nanofluid, permeability of porous medium, $$T\left( {Y,\tilde{t}} \right)$$ is the temperature of the nano-fluid and $$W(Y,\tilde{t})$$ denotes the velocity of nanofluid.

The expressions of $$\overset{\lower0.5em\hbox{$\smash{\scriptscriptstyle\frown}$}}{\rho }_{nf}$$, $$\left( {\overset{\lower0.5em\hbox{$\smash{\scriptscriptstyle\frown}$}}{\rho } \overset{\lower0.5em\hbox{$\smash{\scriptscriptstyle\frown}$}}{\beta } } \right)_{nf}$$,$$\left( {\overset{\lower0.5em\hbox{$\smash{\scriptscriptstyle\frown}$}}{\rho } C_{p} } \right)_{nf} ,\frac{{\kappa_{nf} }}{{\kappa_{f} }},{\text{ and }}\mu_{nf}$$, $$\frac{{\sigma_{nf} }}{{\sigma_{f} }}$$ are;

9 $$\left\{ \begin{gathered} \overset{\lower0.5em\hbox{$\smash{\scriptscriptstyle\frown}$}}{\rho }_{nf} = \ddot{\varphi }\overset{\lower0.5em\hbox{$\smash{\scriptscriptstyle\frown}$}}{\rho }_{s} + (1 - \ddot{\varphi })\overset{\lower0.5em\hbox{$\smash{\scriptscriptstyle\frown}$}}{\rho }_{f} ,\left( {\overset{\lower0.5em\hbox{$\smash{\scriptscriptstyle\frown}$}}{\rho } C_{p} } \right)_{nf} = (1 - \ddot{\varphi })\left( {\overset{\lower0.5em\hbox{$\smash{\scriptscriptstyle\frown}$}}{\rho } C_{p} } \right)_{f} + \ddot{\varphi }\left( {\overset{\lower0.5em\hbox{$\smash{\scriptscriptstyle\frown}$}}{\rho } C_{p} } \right)_{s} , \hfill \\ \left( {\overset{\lower0.5em\hbox{$\smash{\scriptscriptstyle\frown}$}}{\rho } \overset{\lower0.5em\hbox{$\smash{\scriptscriptstyle\frown}$}}{\beta } } \right)_{nf} = (1 - \ddot{\varphi })\left( {\overset{\lower0.5em\hbox{$\smash{\scriptscriptstyle\frown}$}}{\rho } \overset{\lower0.5em\hbox{$\smash{\scriptscriptstyle\frown}$}}{\beta } } \right)_{f} + \ddot{\varphi }\left( {\overset{\lower0.5em\hbox{$\smash{\scriptscriptstyle\frown}$}}{\rho } \overset{\lower0.5em\hbox{$\smash{\scriptscriptstyle\frown}$}}{\beta } } \right)_{s} ,\mu_{nf} = \mu_{f} \left( {1 - \ddot{\varphi }} \right)^{ - 2.5} , \hfill \\ \frac{{\kappa_{nf} }}{{\kappa_{f} }} = \frac{{(\kappa_{s} + 2\kappa_{f} ) - 2\ddot{\varphi }\left( {\kappa_{f} - \kappa_{s} } \right)}}{{(\kappa_{s} + 2\kappa_{f} ) + \ddot{\varphi }\left( {\kappa_{f} - \kappa_{s} } \right)}} = g\left( {\ddot{\varphi }} \right), \hfill \\ \frac{{\sigma_{nf} }}{{\sigma_{f} }} = 1 + \frac{{\left( {\frac{{\sigma_{s} }}{{\sigma_{s} }} - 1} \right)\ddot{\varphi }}}{{\left( {\frac{{\sigma_{s} }}{{\sigma_{s} }} + 2} \right) - \left( {\frac{{\sigma_{s} }}{{\sigma_{s} }} - 1} \right)\ddot{\varphi }}}. \hfill \\ \end{gathered} \right\}$$

Here $$\ddot{\varphi }$$, $$\overset{\lower0.5em\hbox{$\smash{\scriptscriptstyle\frown}$}}{\rho }_{f}$$, $$\overset{\lower0.5em\hbox{$\smash{\scriptscriptstyle\frown}$}}{\rho }_{s}$$, $$C_{p}$$ $$\kappa_{f} ,\kappa_{s} ,\mu_{f}$$ represent the volume fraction of the nanoparticles, the density of the base fluid, the density of the solid particles or the specific heat at constant pressure, the thermal conductivity of the base fluid, the thermal conductivity of the base fluid and the viscosity of the base fluid.

For the prescribed Pde's (Eq. 7 and Eq. 8), the corresponding boundary conditions and initial conditions are as follows;

10 $$W(Y,0) = 0,\;\;T(Y,0) = T_{\infty } \;\;Y \ge 0,$$

11 $$W\left( {0,\tilde{t}} \right) = U_{o} \left( {1 - e^{{ - \gamma \tilde{t}}} } \right),k_{{nf}} \left. {\frac{{\partial T\left( {Y,\tilde{t}} \right)}}{{\partial Y}}} \right|_{{Y = 0}} = - q_{w} ,\tilde{t} > 0,$$

12 $$W(Y,\tilde{t}) = 0,T(Y,\tilde{t}) = T_{\infty } ,\;\;{\text{as}}\;\;Y \to \infty .$$

$q_{w}$ represents heat passing from surface of wall.

Now incorporate the unit less parameters

13 $$\left[ \begin{gathered} Y^{*} = \frac{Y}{N},\tilde{t}^{*} = \frac{{\upsilon_{f} }}{{N^{2} }}\tilde{t},W^{*} = \frac{N}{{\upsilon_{f} }}W,\Theta = \frac{{k_{f} }}{{q_{w} }}(T - T_{\infty } ) \hfill \\ N = \left( {\frac{{k_{f} \upsilon_{f}^{2} }}{{g\overset{\lower0.5em\hbox{$\smash{\scriptscriptstyle\frown}$}}{\beta }_{f} q_{w} }}} \right)^{{^{\frac{1}{4}} }} ,U_{o} = \frac{{\upsilon_{f} }}{N},\gamma^{*} = \frac{{\gamma N^{2} }}{{\upsilon_{f} }}, \hfill \\ \end{gathered} \right]$$

and by neglecting $*$ from Eqs. (7), (8) and from Eqs. (10–12), we get the unit less form given as;

14 $$\vartheta_{1} \frac{{\partial W(Y,\tilde{t})}}{{\partial \tilde{t}}} = \frac{{\partial^{2} W(Y,\tilde{t})}}{{\partial Y^{2} }} - \vartheta_{3}^{ * } W(Y,\tilde{t}) - \vartheta_{4}^{ * } W(Y,\tilde{t}) + \vartheta_{2} \Theta (Y,\tilde{t}),Y,\tilde{t} \ge 0$$

15 $$\vartheta_{3} \frac{{\partial \Theta (Y,\tilde{t})}}{{\partial \tilde{t}}} = \frac{{\partial^{2} \Theta (Y,\tilde{t})}}{{\partial Y^{2} }} - \vartheta_{4} \Theta (Y,\tilde{t}),Y,\tilde{t} \ge 0,$$

16 $$W(Y,0) = 0,\Theta (Y,0) = 0,Y > 0,$$

17 $$W(0,\tilde{t}) = U_{o} \left( {1 - e^{{ - \gamma \tilde{t}}} } \right),g(h,\ddot{\varphi })\left. {\frac{{\partial \Theta (Y,\tilde{t})}}{{\partial Y}}} \right|_{{Y = 0}} = - 1,\tilde{t} > 0,$$

18 $$W(Y,\tilde{t}) = 0,\Theta (Y,\tilde{t}) \to 0,\;\;{\text{as}}\;\;Y \to \infty .$$

$\vartheta_{1}$, $\vartheta_{2}$, $\vartheta_{3}^{ * }$, $\vartheta_{4}^{ * }$, $\vartheta_{3}$, and $\vartheta_{4}$ are values in the equations before which can be expressed as;

19 $$\left\{ \begin{gathered} \vartheta_{1} = \frac{1}{{p(\ddot{\varphi })}}\left( {(1 - \ddot{\varphi }) + \ddot{\varphi }\frac{{\overset{\lower0.5em\hbox{$\smash{\scriptscriptstyle\frown}$}}{\rho }_{s} }}{{\overset{\lower0.5em\hbox{$\smash{\scriptscriptstyle\frown}$}}{\rho }_{f} }}} \right),\vartheta_{2} \frac{1}{{p(\ddot{\varphi })}}\left( {(1 - \ddot{\varphi }) + \ddot{\varphi }\frac{{(\overset{\lower0.5em\hbox{$\smash{\scriptscriptstyle\frown}$}}{\rho } \overset{\lower0.5em\hbox{$\smash{\scriptscriptstyle\frown}$}}{\beta } )_{s} }}{{(\overset{\lower0.5em\hbox{$\smash{\scriptscriptstyle\frown}$}}{\rho } \overset{\lower0.5em\hbox{$\smash{\scriptscriptstyle\frown}$}}{\beta } )_{f} }}} \right), \hfill \\ \vartheta *_{3} = \frac{{\overset{\lower0.5em\hbox{$\smash{\scriptscriptstyle\frown}$}}{\sigma }_{f} B_{o}^{2} N^{2} }}{{\overset{\lower0.5em\hbox{$\smash{\scriptscriptstyle\frown}$}}{\rho }_{f} \upsilon_{f} p(\ddot{\varphi })}} = \frac{{\sigma_{nf} }}{{\sigma_{f} }}\frac{{M^{2} }}{{p(\ddot{\varphi })}}, \, \vartheta *_{4} = \frac{{N^{2} }}{K} = K_{p} \hfill \\ \vartheta_{3} = \frac{\Pr }{{g(\ddot{\varphi })}}\left( {(1 - \ddot{\varphi }) + \ddot{\varphi }\frac{{(\rho C_{p} )_{s} }}{{(\rho C_{p} )_{f} }}} \right),Pr = \frac{{\left( {\overset{\lower0.5em\hbox{$\smash{\scriptscriptstyle\frown}$}}{\mu } C_{p} } \right)_{f} }}{{\overset{\lower0.5em\hbox{$\smash{\scriptscriptstyle\frown}$}}{k}_{f} }},\vartheta_{4} = \frac{{\upsilon_{f} Q^{ * } }}{{g(\ddot{\varphi })\sqrt {g\overset{\lower0.5em\hbox{$\smash{\scriptscriptstyle\frown}$}}{\beta }_{f} \overset{\lower0.5em\hbox{$\smash{\scriptscriptstyle\frown}$}}{k}_{f} } q_{w} }} \hfill \\ \end{gathered} \right\},$$

where $\Pr ,M,K_{p}$ are respectively represents the Prandtl number, the Magnetic factor and the inverse permeability.

To obtain a fractional model, we include the Caputo-Fabrizio time derivative in Eqs. (7) and (8):

20 $$\vartheta_{1} {}^{CF}\delta_{t}^{{\hat{\alpha }}} W(Y,\tilde{t}) = \frac{{\partial^{2} W(Y,\tilde{t})}}{{\partial Y^{2} }} - \vartheta_{3}^{ * } W(Y,\tilde{t}) - \vartheta_{4}^{ * } W(Y,\tilde{t}) + \vartheta_{2} \Theta (Y,\tilde{t}),0 < \hat{\alpha } \le 1,Y,\tilde{t} \ge 0,$$

21 $$\vartheta_{3} {}^{CF}\delta_{t}^{{\hat{\beta }}} \Theta (Y,\tilde{t}) = \frac{{\partial^{2} \Theta (Y,\tilde{t})}}{{\partial Y^{2} }} - \vartheta_{4} \Theta (Y,\tilde{t}),0 < \hat{\beta } \le 1,Y,\tilde{t} \ge 0.$$

The Caputo-Fabrizio time fractional derivative and their Laplace transform are given by;

22 $${}^{CF}\delta_{\tau }^{\chi } Z(Y,\tau ) = \frac{1}{1 - \tau }\int\limits_{0}^{\tau } {\frac{\partial Z(Y,r)}{{\partial r}}} \, e^{{\left( { - \frac{\chi (\tau - \chi )}{{1 - \chi }}} \right)}} dr,0 < \chi < 1,$$

23 $$L\left\{ {{}^{CF}\delta_{\tau }^{\chi } Z(Y,\tau )} \right\} = \frac{{r\overline{Z}(Y,r) - \overline{Z}(Y,0)}}{(1 - \chi )r + \chi }.$$

‘L’ denotes the LT.

## Solution of the problem

### Temperature field

Taking LT on (21) and make use of respective transformed ICs and BCs along with Eq. (23), we obtained

24 $$\frac{{\partial^{2} \overline{\Theta } (Y,r)}}{{\partial Y^{2} }} = \left[ {\frac{\eta q + \chi }{{r + \psi }}} \right]\overline{\Theta } (Y,r)$$

where

$$\eta = \frac{{\vartheta_{3} }}{{(1 - \hat{\beta })}} + \vartheta_{4} ,\chi = \frac{{\vartheta_{4} \hat{\beta }}}{{1 - \hat{\beta }}},\psi = \frac{{\hat{\beta }}}{1 - v},\hat{\beta } \in (0,1),$$

$r$ represents the Laplace frequency and $\beta$ is the fractional parameter.

25 $$\left. {\frac{{\partial \bar{\Theta }(Y,r)}}{{\partial Y}}} \right|_{{Y = 0}} = \frac{{ - 1}}{{g(\ddot{\varphi })r}},\bar{\Theta }(Y,r) \to 0,\;\;Y \to \infty .$$

For the solution of Eq. (24) and utilizing Eq. (25), we get

26 $$\overline{\Theta }\left( {Y,r} \right) = \frac{1}{{g(\ddot{\varphi })}}\frac{1}{r}\overline{\Omega } (Y,r;\eta ,\chi ,\psi ),$$

where

27 $$\overline{\Omega } (Y,r;\eta ,\chi ,\psi ) = \frac{1}{r}\frac{1}{{\sqrt {\frac{\eta r + \chi }{{r + \psi }}} }}e^{{ - Y\sqrt {\frac{\eta r + \chi }{{r + \psi }}} }} = \frac{1}{r}\frac{1}{{\sqrt {d_{{\hat{\beta }}} (r)} }}e^{{ - Y\sqrt {d_{{\hat{\beta }}} (r} )}} ,$$

also

28 $$\, d_{{\hat{\beta }}} (r) = \frac{\eta r + \chi }{{r + \psi }} = \frac{{\left[ {\vartheta_{3} + (1 - \hat{\beta })\vartheta_{4} } \right] + \vartheta_{4} \hat{\beta }}}{{(1 - \hat{\beta })r + \vartheta_{4} }},$$

Now we have to find $\overline{\Omega }$ which is solved by using Laplace inverse over $\overline{\Omega }$ but the given function is not a simple function, it is a compound function and can be defined as;

If $\overline{F}(r)$ be a function then the Laplace inverse $\overline{F}(r)$ of is $F(\tilde{t})$. Then the LIT of $F(d(r))$ is represented by;

$$\, L^{ - 1} \{ F(d(q))\} = \int\limits_{0}^{\infty } {F(m)s(m,\tilde{t})} dm, \;\; \text{and}$$

29 $$s(m,\tilde{t}) = L^{ - 1} \left\{ {e^{( - md(r))} } \right\}$$

Taking LIT to Eq. (28) and utilizing Faltung product present in Eq. (23), here $\overline{F}(r) = \left( {\frac{1}{\sqrt r }} \right)e^{ - Y\sqrt r }$ and $d(r) = d_{\beta } (r)$, we acquired the Laplace inverse of $\overline{\Omega }(Y,r;\eta ,\chi ,\psi )$, we have

30 $$\Omega (Y,\tilde{t};\eta ,\chi ,\psi ) = \left\{ \begin{gathered} \frac{1}{\sqrt \pi }H(\tilde{t})\int\limits_{0}^{\infty } {\frac{1}{\sqrt \pi }} e^{{\left( {\frac{{ - Y^{2} }}{4m} - \eta m} \right)}} dm + \hfill \\ \sqrt {\frac{\eta \psi - \chi }{\pi }} \int\limits_{0}^{\infty } {e^{{\left( {\frac{{ - Y^{2} }}{4m} - \eta m} \right)}} } \times \hfill \\ \int\limits_{0}^{\infty } {\frac{{e^{ - \psi n} }}{\sqrt n }J_{1} \left( {2\sqrt {(\eta \psi - \chi )mn} } \right)dndm} \hfill \\ \end{gathered} \right\},$$

The unit step Heaviside function *H(t)* and the Bessel modified function of order one and first types are expressed in the preceding equation. The more reliable form of Eq. (30) is given below;

31 $$\Omega (Y,\tilde{t};\eta ,\chi ,\psi ) = \frac{1}{\sqrt \eta }H(\tilde{t})e^{ - Y\sqrt \eta } + \sqrt {\frac{\eta c - b}{\pi }} \int\limits_{0}^{\infty } {e^{{\left( {\frac{{ - Y^{2} }}{4m} - \eta m} \right)}} } \times \int\limits_{0}^{{\tilde{t}}} {\frac{{e^{ - \psi n} }}{\sqrt n }J_{1} \left( {2\sqrt {(\eta \psi - \chi )mn} } \right)dndm} ,$$

Applying LIT upon Eq. (26) we acquired

32 $$\Theta (Y,\tilde{t}) = \frac{1}{{g(h,\ddot{\varphi })}}\frac{1}{r}\Omega (Y,\tilde{t};\eta ,\chi ,\psi ).$$

### Nusslet number

The Nusselt number Nu, is taken from^23^,

33 $$Nu\left( r \right) = \frac{{Nq_{w} }}{{k_{f} (T_{w} - T_{\infty } )}} = \frac{1}{{\Theta (Y,\tilde{t})}}|_{Y = 0} = \frac{1}{{L^{ - 1} \{ \overline{\Theta } (0,r)\} }},$$

34 $$Nu\left( {\tilde{t}} \right) = \frac{{g(r,\ddot{\varphi })}}{{\Omega (0,\tilde{t};\eta ,\chi ,\psi )}},$$

The expression $\Omega (0,\tilde{t};\eta ,\chi ,\psi )$ has been acquired by using

35 $$\Omega (0,\tilde{t};\eta ,\chi ,\psi ) = L^{ - 1} \left\{ \begin{gathered} \frac{1}{r}\sqrt {\frac{r + \psi }{{\eta r + \chi }}} = \frac{1}{\sqrt \eta }e^{{\left( {\frac{{ - (\eta \psi + \chi )\tilde{t}}}{2\eta }} \right)}} \times J_{o} \left( {\frac{{ - (\eta \psi + \chi )\tilde{t}}}{2\eta }} \right)H(\tilde{t}) + \hfill \\ \frac{\psi }{\sqrt \eta } \times \int\limits_{0}^{{\tilde{t}}} {e^{{\left( {\frac{ - (\eta \psi + \chi )m}{{2\eta }}} \right)}} J_{o} \left( {\frac{ - (\eta \psi + \chi )m}{{2\eta }}} \right)} dm. \hfill \\ \end{gathered} \right\}$$

To find the thermal boundary layer thickness in term of fractional derivative. We will integrate thermal layer Eq. (24) from $Y \to 0$ to $Y \to \infty$

36 $$\Phi_{T} (\tilde{t}) = \int\limits_{0}^{{\Phi_{1T} }} {\Theta (Y,\tilde{t})dY,}$$

By utilizing the ICs and BCs in Eqs. (17) and (18), we acquired

37 $$\vartheta_{3} {}^{CF}\delta_{{\tilde{t}}}^{{\hat{\beta }}} \Phi_{T} (\tilde{t}) + \vartheta_{4} \delta_{{\tilde{t}}}^{{\hat{\beta }}} \Phi_{T} (\tilde{t}) = \frac{1}{{g(h,\ddot{\varphi })}},$$

After solving Eq. (37) and using respective ICs and BCs, we acquired

38 $$\Phi_{T} \left( {\tilde{t}} \right) = \frac{1}{{\vartheta_{4} g(h,\ddot{\varphi })}}\left\{ {1 - \frac{{\vartheta_{3} }}{{\vartheta_{3} + (1 - \hat{\beta })\vartheta_{4} }}e^{{\left( {\frac{{ - \vartheta_{4} \hat{\beta }\hat{t}}}{{\vartheta_{3} + (1 - \hat{\beta })\vartheta_{4} }}} \right)}} } \right\},$$

For $\hat{\beta } \to 1$ (integer order derivative), Eq. (38) becomes.

39 $$\Phi_{T} \left( {\tilde{t}} \right) = \frac{1}{{\vartheta_{4} g(h,\ddot{\varphi })}}\left\{ {1 - e^{{\left( {\frac{{ - \vartheta_{4} \tilde{t}}}{{\vartheta_{3} }}} \right)}} } \right\}.$$

### Dimensional less velocity and skin Friction coefficient

Taking LT of Caputo-Fabrizio derivative of Eq. (23) upon Eq. (20) and their respective ICs and BCs and incorporate Eq. (27), we acquired

40 $$\frac{{\partial^{2} \overline{W} (Y,r)}}{{\partial Y^{2} }} = \frac{{sr\overline{W} (Y,r)}}{r + j} + \vartheta_{3}^{ * } \overline{W} (Y,r) + \vartheta_{4}^{ * } \overline{W} (Y,r) - \vartheta_{2} \frac{1}{{g(\ddot{\varphi })r}}\frac{1}{{\sqrt {d_{{\hat{\beta }}} (r)} }}e^{{ - Y\sqrt {d_{{\hat{\beta }}} (r} )}}$$

Where

$$s = \frac{{\vartheta_{1} }}{{1 - \hat{\alpha }}}{, }j{ = }\frac{{\hat{\alpha }}}{{1 - \hat{\alpha }}},$$

After solving Eq. (40) and using ICs and BCs, we acquired

41 $$\overline{W} (Y,r) = \left\{ \begin{gathered} \frac{\gamma }{r(r + \gamma )}\overline{\zeta } (Y,r;s,j,\vartheta_{3}^{ * } ,\vartheta_{4}^{ * } ) + a_{2} \overline{D} \left( r \right))\overline{\Theta } (Y,r) \hfill \\ \, - a_{2} \overline{\Theta } (0,r)\overline{D} (r)\overline{\zeta } (Y,r;s,j,\vartheta_{3}^{ * } ,\vartheta_{4}^{ * } ) \hfill \\ \end{gathered} \right\},$$

$$\overline{\zeta } (Y,r;s,j,\vartheta_{3}^{ * } ,\vartheta_{4}^{ * } ) = e^{{ - Y\sqrt {U_{{\hat{\alpha }}} (r)} }} ,{\text{ Where }}U_{{\hat{\alpha }}} (r) = \frac{sr}{{r + j}} + \vartheta_{3}^{ * } + \vartheta_{3}^{ * } ,$$

and

42 $$\begin{gathered} \overline{D} (r) = \left\{ \begin{gathered} \frac{(r + \psi )(r + j)}{{(s + \vartheta_{3}^{ * } + \vartheta_{4}^{ * } - \eta )r^{2} - r(\eta j + \chi - s\psi - \vartheta_{3}^{ * } \psi + \vartheta_{4}^{ * } - j\psi \vartheta_{3}^{ * } - \vartheta_{4}^{ * } j) - (j\chi - \vartheta_{3}^{ * } j\psi - \vartheta_{4}^{ * } j\psi )}} \hfill \\ = \frac{1}{s - \eta }\left( {1 + \frac{{l_{11} }}{{r - q_{1} }} - \frac{{l_{12} }}{{r - q_{2} }}} \right){ , } \quad s \ne \eta . \, \hfill \\ \end{gathered} \right\} \hfill \\ \, \hfill \\ \end{gathered}$$

where $$l_{11} = \frac{{(q_{1} + \psi )(q_{1} + j)}}{{q_{1} - q_{2} }},l_{12} = \frac{{(q_{2} + \psi )(q_{2} + j)}}{{q_{1} - q_{2} }}.$$

And

43 $$q_{1,2} = \frac{{\left\{ \begin{gathered} \eta j + \chi - s\psi - \vartheta_{3}^{ * } \psi - \vartheta_{4}^{ * } \psi - \vartheta_{3}^{ * } j - \vartheta_{4}^{ * } j \pm \hfill \\ \sqrt {(\eta j + \chi - s\psi - \vartheta_{3}^{ * } \psi - \vartheta_{4}^{ * } \psi - \vartheta_{3}^{ * } j - \vartheta_{4}^{ * } j)^{2} + 4(\chi j - \vartheta_{3}^{ * } j\psi - \vartheta_{4}^{ * } j\psi )(s + \vartheta_{3}^{ * } + \vartheta_{4}^{ * } - \eta )} \hfill \\ \end{gathered} \right\}}}{{2(s + \vartheta_{3}^{ * } + \vartheta_{4}^{ * } - \eta )}}$$

are the polynomial roots

44 $$p(r) = (s + \vartheta_{3}^{ * } + \vartheta_{3}^{ * } - \eta )r^{2} - r(\eta j + \chi - \psi s - \vartheta_{3}^{ * } \psi + \vartheta_{4}^{ * } - \vartheta_{3}^{ * } \psi j - \vartheta_{4}^{ * } \psi ) - (\eta j - \vartheta_{3}^{ * } \psi j - \vartheta_{4}^{ * } \psi j)$$

Applying Laplace inverse transform to Eq. (41) using Eq. (29) i.e. Eq. of compound function. with $E(r) = e^{ - Y\sqrt r } {\text{ and }}U_{{\hat{\alpha }}} (r) = \frac{sr}{{r + j}} + \vartheta_{3}^{ * } + \vartheta_{3}^{ * } ,$ we acquired

45 $$\zeta (Y,\tilde{t};s,j,\vartheta_{3}^{ * } ,\vartheta_{4}^{ * } ) = \delta (\tilde{t})e^{{ - Y\sqrt {s + \vartheta_{3}^{ * } + \vartheta_{4}^{ * } } }} + \zeta_{o} (Y,\tilde{t};s,j,\vartheta_{3}^{ * } ,\vartheta_{4}^{ * } ),$$

where

46 $$\zeta_{o} \left( {Y,\tilde{t};s,j,\vartheta_{3}^{ * } ,\vartheta_{4}^{ * } } \right) = \frac{{Y\sqrt {sj} e^{{ - j\tilde{t}}} }}{{2\sqrt {\pi \tilde{t}} }}\int\limits_{0}^{\infty } {\frac{{e^{{^{{\left( {\frac{{ - Y^{2} }}{4x}} \right. - x\left( {s + \vartheta_{3}^{ * } + \vartheta_{3}^{ * } } \right)}} }} }}{x} \times J_{1} \left( {2\sqrt {sjx\tilde{t}} } \right)dx.}$$

The LIT of $\overline{D}(r)$, present in Eq. (42) is,

47 $$D(\tilde{t}) = \frac{1}{s - \eta }\left( {\delta (\tilde{t}) + l_{11} e^{{q_{1} \tilde{t}}} - l_{12} e^{{q_{2} \tilde{t}}} } \right) \,$$

Applying the Laplace inverse transformation on Eq. (41) and Faltung theorem, we acquired

48 $$\begin{aligned} W(Y,\tilde{t}) & = \left( {1 - e^{{ - \gamma \tilde{t}}} } \right)e^{{ - Y\sqrt {s + \vartheta_{3}^{ * } + \vartheta_{4}^{ * } } }} + \vartheta_{2} \int\limits_{0}^{{\tilde{t}}} {\left( {1 - e^{{ - \gamma (\tilde{t} - k)}} } \right)\zeta_{o} (Y,k;s,\vartheta_{3}^{ * } ,\vartheta_{4}^{ * } )dk + \vartheta_{2} \int\limits_{0}^{{\tilde{t}}} {D(\tilde{t} - k)\Theta (Y,k)dk} } \hfill \\ & \quad - \vartheta_{2} \int\limits_{0}^{{\tilde{t}}} {\int_{0}^{k} {\Theta (0,\tilde{t} - k)D(k - \varsigma )\zeta_{o} (Y,\varsigma ;s,\vartheta_{3}^{ * } ,\vartheta_{4}^{ * } )d\varsigma dk.} } \, \hfill \\ \end{aligned}$$

Skin friction coefficient is a basic physical quantity of relevance that is defined as

49 $$C_{f} = \left\{ \begin{gathered} \frac{{\hat{\tau }_{w} }}{{\hat{\rho }_{f} ({{\hat{\upsilon }_{f} } \mathord{\left/ {\vphantom {{\hat{\upsilon }_{f} } N}} \right. \kern-\nulldelimiterspace} N})^{2} }} = p(\ddot{\varphi })\frac{{\partial^{2} W(Y,\tilde{t})}}{{\partial Y^{2} }}|_{Y = 0} = p(\ddot{\varphi })L^{ - 1} \left( {\frac{{\partial^{2} W(Y,\tilde{t})}}{{\partial Y^{2} }}|_{Y = 0} } \right) \hfill \\ = p(\ddot{\varphi })L^{ - 1} \left( {\vartheta_{2} \overline{\Theta } {(0,}r{)}\overline{D} {(}r{)}\overline{Q} {(}r{) - }\frac{{\vartheta_{2} }}{{g(h,\ddot{\varphi })}}\frac{1}{r}\overline{D} {(}r{)} - \frac{\gamma }{r(r + \gamma )}\overline{Q} {(}r{)}} \right) \hfill \\ \end{gathered} \right\},$$

where

50 $$Q(r) = \sqrt {\frac{sr}{{r + j}} + \vartheta_{3}^{ * } + \vartheta_{4}^{ * } } \,$$

The LIT of skin friction coefficient is;

51 $$C_{f} = p(\ddot{\varphi })\left\{ {\vartheta_{2} \int\limits_{0}^{{\tilde{t}}} {\int\limits_{0}^{k} {D{(}k - \varsigma {)}\Theta {(0,}\tilde{t} - k{)}Q{(}\varsigma } } {)}d\varsigma dk - \frac{{\vartheta_{2} }}{{g(h,\ddot{\varphi })}} \times \int\limits_{0}^{{\tilde{t}}} {D{(}k{)}dk} - \int\limits_{0}^{{\tilde{t}}} {Q(k)(1 - e^{{ - \gamma (\tilde{t}{ - }k)}} )dk} } \right\},$$

With

52 $$Q(\tilde{t}) = \sqrt \vartheta \left( {\frac{\vartheta j - \chi }{{2\vartheta }}J_{o} \left( {\frac{\vartheta j - \chi }{{2\vartheta }}\tilde{t}} \right) + \delta (\tilde{t})} \right)e^{{\left( {\frac{ - \vartheta j + \chi }{{2\vartheta }}\tilde{t}} \right)}} + \frac{\chi - \vartheta j}{{2\sqrt \vartheta }}J_{o} \left( {\frac{\vartheta j - \chi }{{2\vartheta }}\tilde{t}} \right)e^{{\left( {\frac{ - \vartheta j + \chi }{{2\vartheta }}\tilde{t}} \right)}} .$$

## Numerical results and discussions

**Figure 2:**
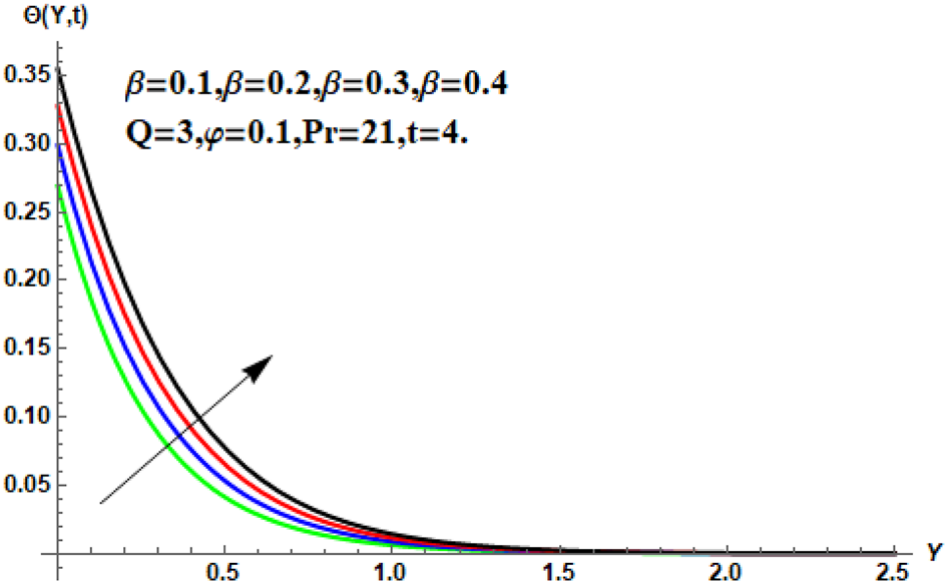
Temperature profile for different values of $\beta$.

**Figure 3:**
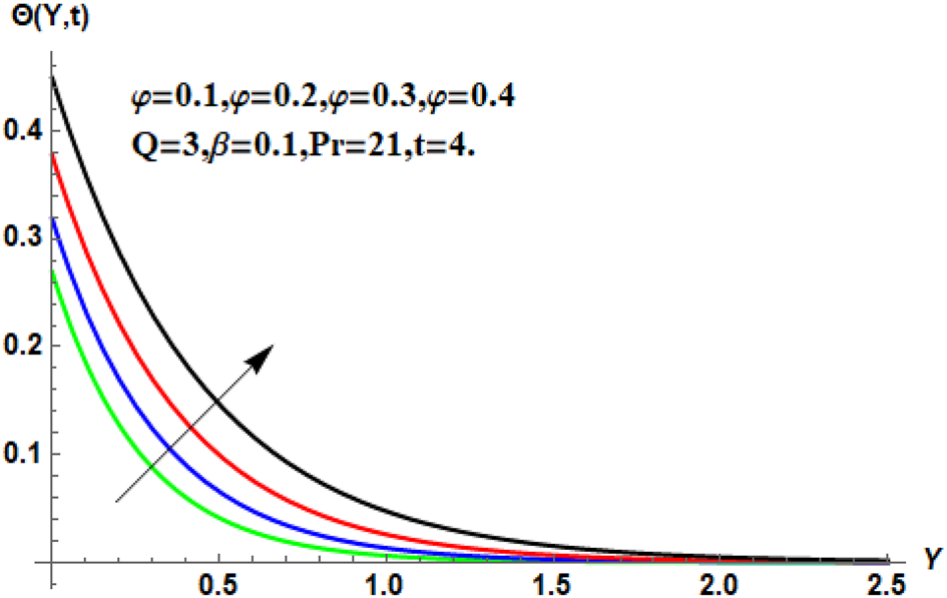
Temperature profile for different values of $\ddot{\varphi }$.

**Figure 4:**
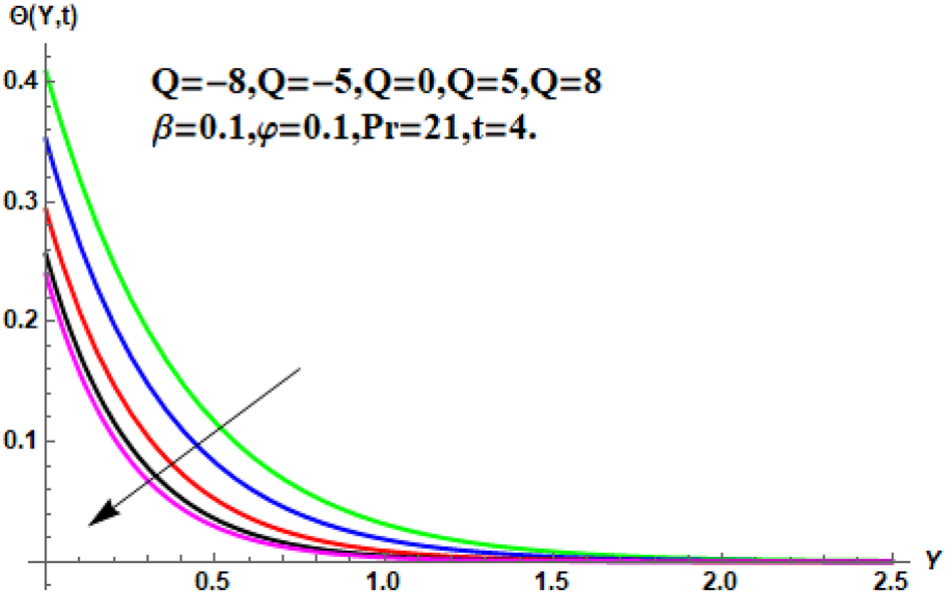
Temperature profile for different values of $Q$.

**Figure 5:**
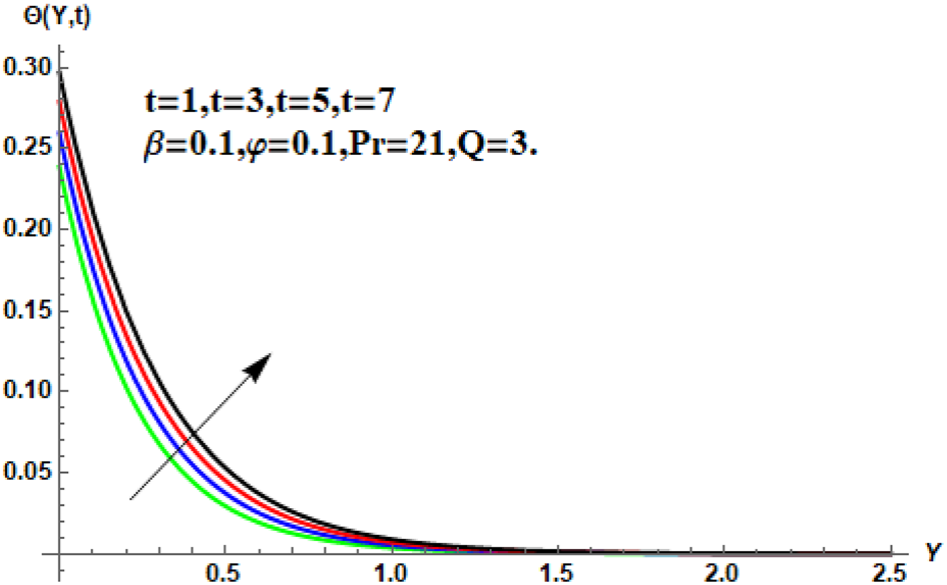
Temperature profile for different values of $t$.

**Figure 6:**
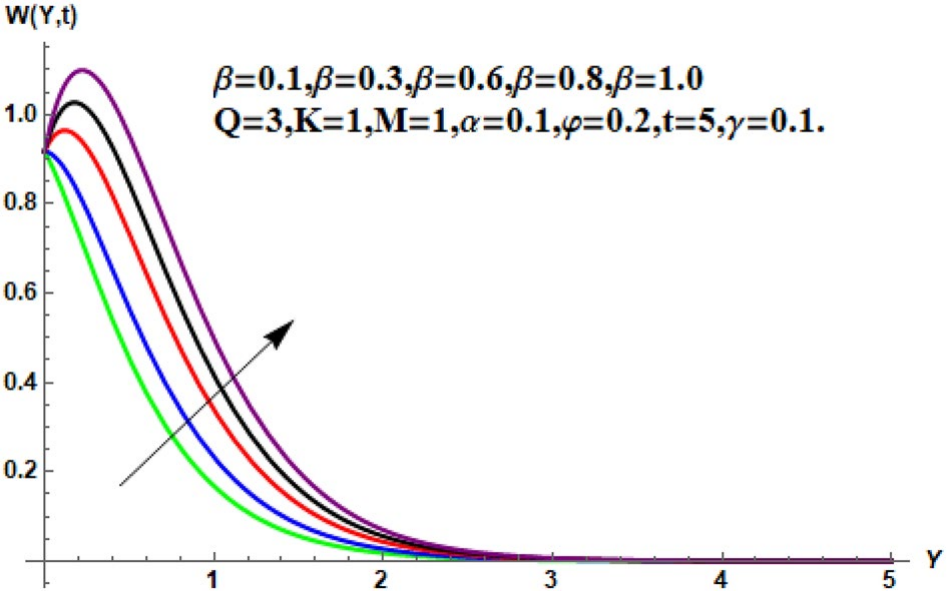
Velocity profile for different values of $\beta$.

**Figure 7:**
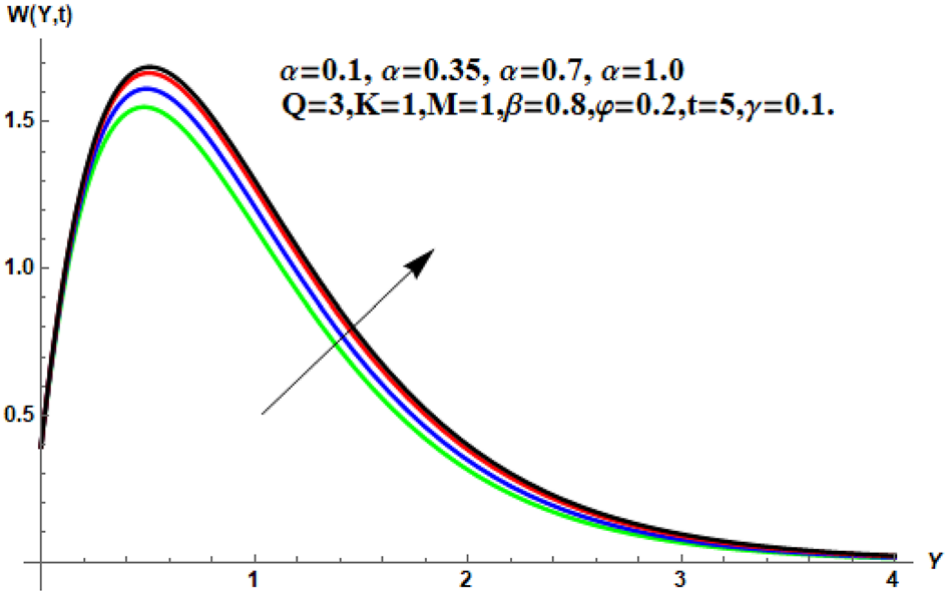
Velocity profile for different values of $\alpha$.

**Figure 8:**
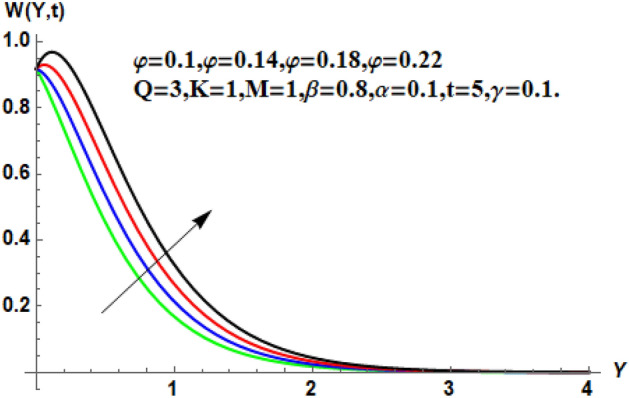
Velocity profile for different values of $\ddot{\varphi }$.

**Figure 9:**
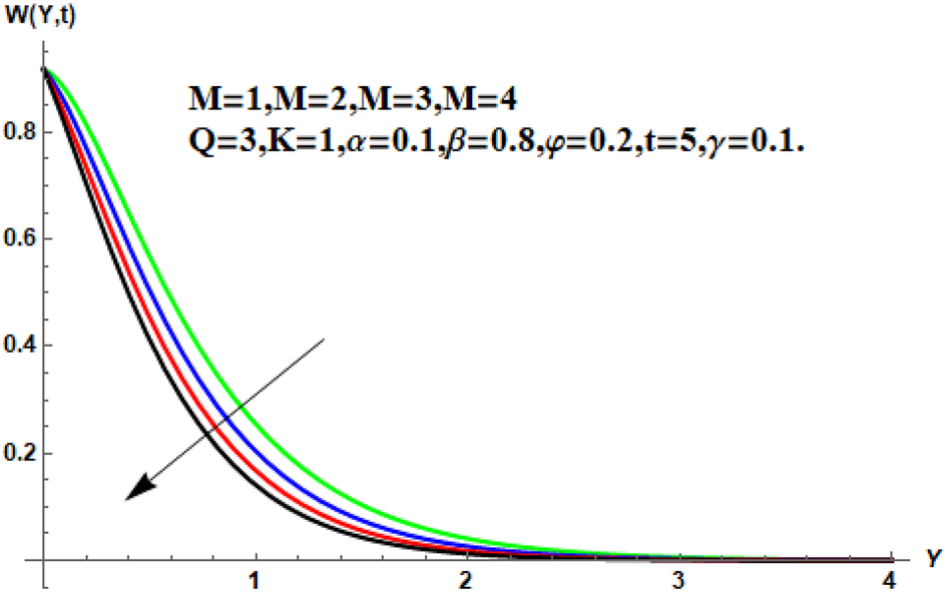
Velocity profile for different values of $M$.

**Figure 10:**
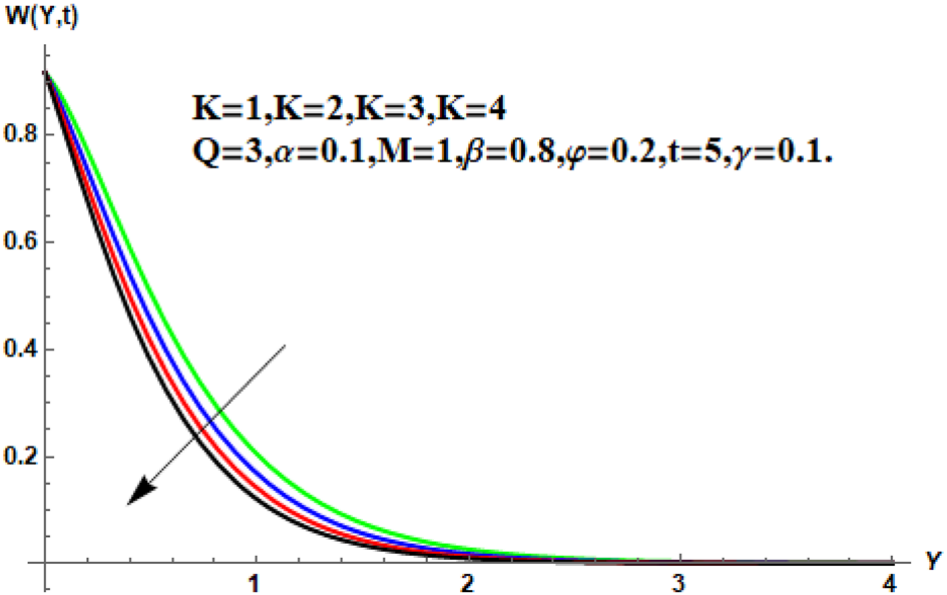
Velocity profile for different values of $K$.

**Figure 11:**
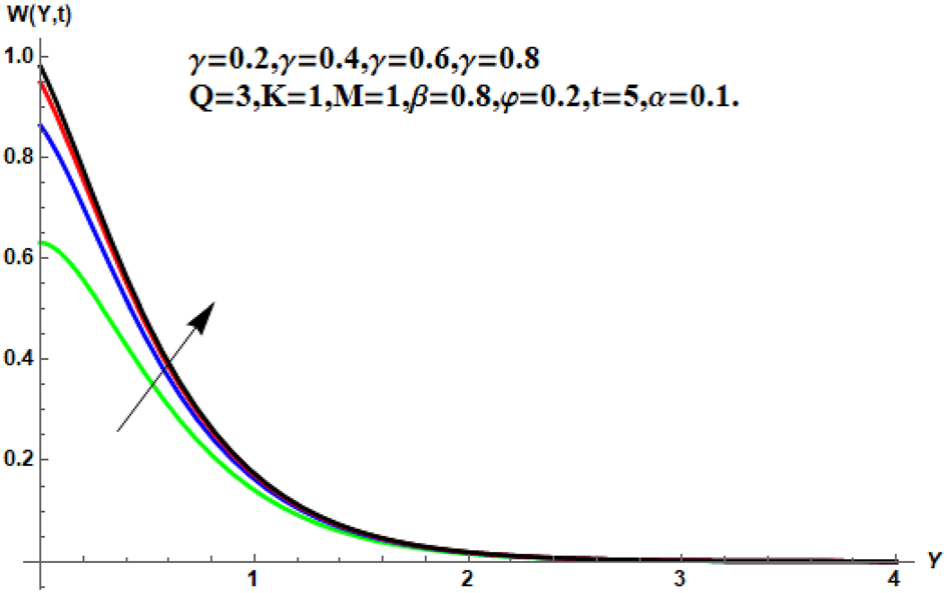
Velocity profile for different values of $\gamma$.

**Figure 12:**
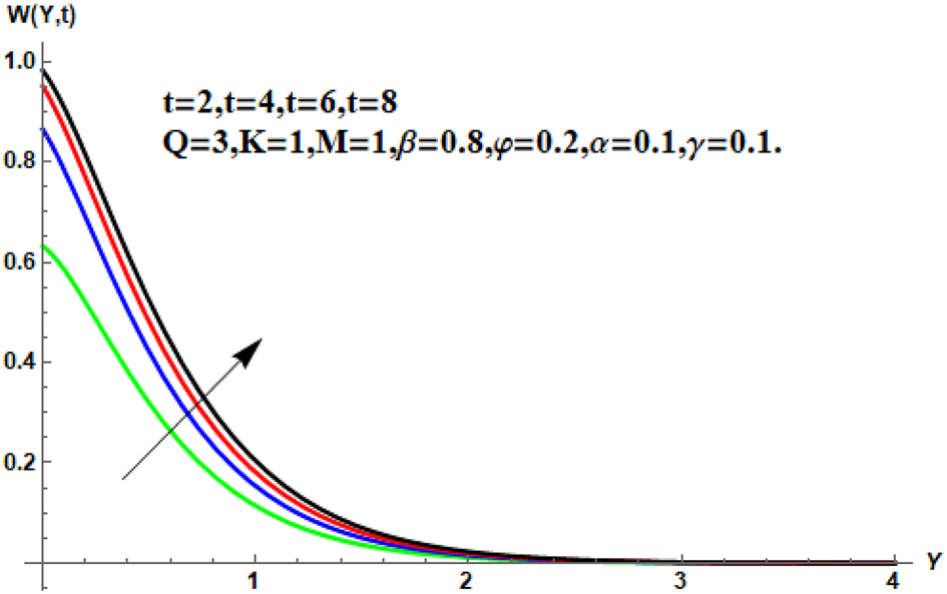
Velocity profile for different values of $t$.

In the following section, a detailed graphical description of the results obtained in the previous section is given. Figures 2, 3, 4 and 5 show the behavior of various parameters with respect to the temperature curve. Figure 2 shows the physical observation of the fractional parameter on the temperature field. It shows that the temperature of nanofluids increases with the increase of the estimated fractional parameter. Physically, this behavior is due to the kernel of the fractional operator. The kernel studied the memory of the function and is capable of impectvely capturing the memory effect through the process. Thus, temperature of nanofluid elevates. From Fig. 3 it can be seen that the temperature of the nanofluid increases with increasing value of the volume fraction physically, this result is due to the high thermal conductivity of CNTs, which causes the thermal conductivity of the base fluid to increase when CNTs are added to it. Consequently, the temperature profile grows. This result highlights the importance of nanoparticles in the heating and cooling process. Figure 4 shows the temperature of the outline when the heat injector or the heat sink is is associated to the system. The temperature field falls with the intensifying estimations of. In associated graph, represents heat consumption, represents heat injection and represents that no heat is consumed or supplied. Physically, the addition of heat means an increase in the temperature of the nanofluid, while the consumption of heat means a decrease in the temperature of the nanofluid. In this process, heat is consumed because the temperature is lowered. Figure 5 shows the transient effect on the temperature curve. The nanofluid temperature curve increases as the time period increases. The temperature of the nanofluid is high near the plate and finally reaches zero asymptotically away from the plate. Figures 6, 7, 8, 9, 10, 11 and 12 show the characteristics of various relevant parameters on the velocity contour. Figure 6 describes the effect of the fractional parameter on velocity. It is worth noted that the nanofluid’s velocity boost with accelerating the fractional parameter. Physically, it is due to the higher value of momentum boundary layer, the velocity is boosted.

**Figure 13:**
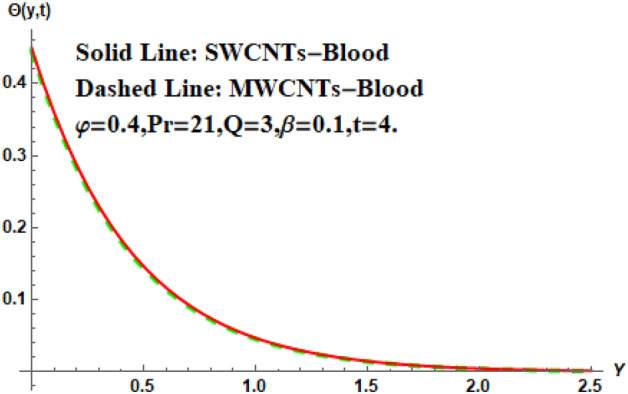
Analysis of SWCNTs and MWCNTs on $\Theta \left( {Y,t} \right).$

**Figure 14:**
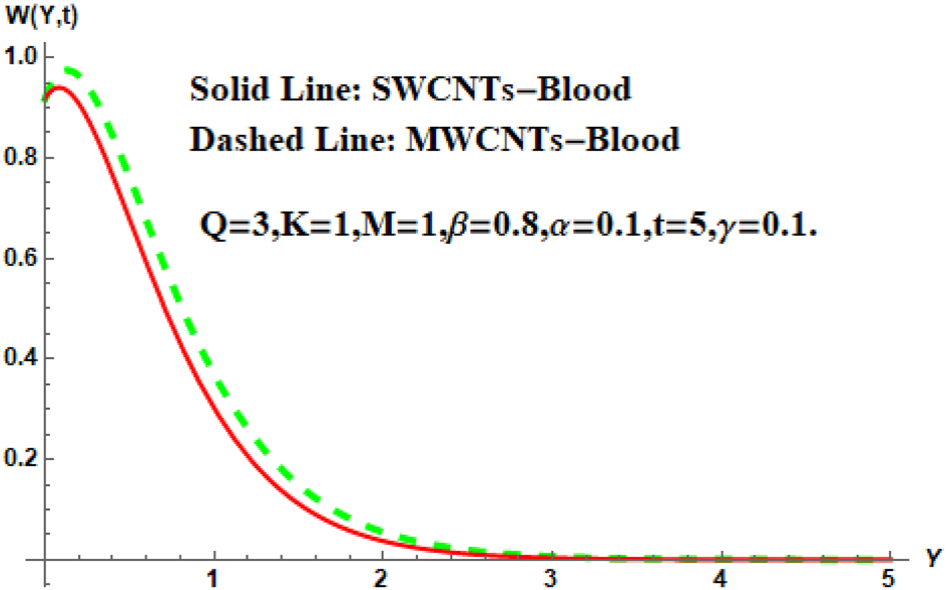
Analysis of SWCNTs and MWCNTs on $W\left( {Y,t} \right).$

**Figure 15:**
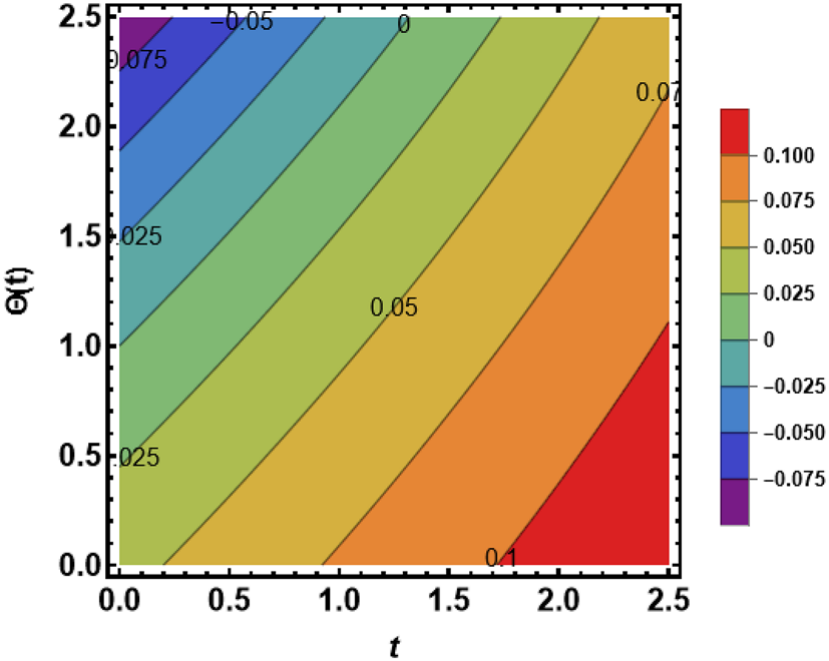
Analysis of $\beta$ on thermal boundary layer thickness.

Figure 7 shows the effects of the fractional parameter on the velocity contour. The higher the fractional parameter is estimated, the higher the velocity of the nanofluids. Figure 8 shows the behavior of the volume fractional parameter on the velocity contour. From Fig. 8, it can be seen that the velocity and momentum of the boundary layer of the nanofluids increases. Physically, the resistance between the particles of the nanofluid is low due to the higher temperature, so the velocity increases. This is also due to the fact that the suspension of CNTs in the base fluid reduces the viscous forces and leads to an increase in the momentum boundary layer. Figure 9 shows the characteristics of the magnetic factor on the velocity sketch. The velocity of the nanofluid decreases at a higher value of the magnetic factor. This is because the magnetic field acts on electrically isolated nanofluids, which behave as a source for generating Lorentz drag forces. Because of these drag forces, the velocity of the nanofluids decreases. As the fluids move away from the plate, the Lorentz force weakens and the fluid comes to rest. Figure 10 shows the influence of the inverse permeability parameter on the velocity of the nanofluid. The momentum boundary layer thickness and velocity decreases with a larger estimate of permeability parameters . Physically, due to the high porosity of the medium, the resistance in the nanofluid particles increases, causing the velocity to decrease. In Fig. 11, the influence of on the velocity of the nanofluid is shown. It can be seen that the velocity increases as the estimate of increases. The velocity is initially higher, later it asymptotically approaches zero. Physically, this happens because there is inverse relation between and viscous forces. As we elevate the estimation of, the viscous forces reduce. As a result, velocity of nanofluid rises. Figure 12 shows that the velocity of nanofluids increases with increasing time value. The momentum boundary layer is raised for a higher estimate of the transient effects. Figure 13 shows the effect of SWCNTs and MWCNTs on the temperature distribution. The temperature of SWCNTs is higher than that of MWCNTs due to the high thermal conductivity of SWCNTs. Figure 14 shows the comparison between the velocity of SWCNTs and MWCNTs. It shows that the velocity of MWCNTs is larger than that of SWCNTs. Figure 15 is the contour plot for the thickness of the thermal layer. The thickness of the thermal boundary layer decreases as we increase the estimates of the fractional parameters Table 2 shows the properties of various relevant parameters on the Nusselt number of SWCNTs and MWCNTs. It can be seen that the heat transport rate increases with the increase of heat source/sink and the time while decrease occurs against fractional parameter and volume fraction From Table 3, it can be seen that the skin fraction (drag forces) increases with the increase in the fractional parameter while the function against the other fractional parameter for both SWCNTs and MWCNTs. Similarly, skin friction is dominant with the increasing value of the magnetic factor, permeability parameter and heat source or sink. Moreover, the drag forces are de-escalates with the escalation of volume fraction time and furthermore, the skin fraction of MWCNTs is lower than the SWCNTs.

**Table 2:** Demonstrated the influence of various parameters on Nusslet number of both SWCNTs and MWCNTs.

$\beta$	$Q$	$\Pr$	$\ddot{\varphi }$	$t$	$Nu\left( {SWCNTs} \right)$	$Nu\left( {MWCNTs} \right)$
0.1	3.0	21	0.1	4.0	− 545.658554	- 546.832866
0.5					− 706.781229	− 708.382133
1.0					− 2585.680572	− 2591.940846
	− 10				− 384.950000	− 546.832865
	0				− 383.294381	− 384.949666
	10				− 112.772886	− 114.019810
			0.1		− 545.658554	− 546.832866
			0.2		− 615.597709	− 616.643750
			0.3		− 759.999526	− 762.116157
				3.0	− 689.453825	− 694.570271
				4.0	− 545.658554	− 546.832866
				5.0	− 349.131762	− 349.883078

**Table 3:** Depicts the impact of various pertinent parameters on skin fraction of both SWCNTs and MWCNTs.

$\alpha$	$\beta$	$\gamma$	$\ddot{\varphi }$	$M$	$K$	$Q$	$t$	$Cf\left( {SWCNTs} \right)$	$Cf\left( {MWCNTs} \right)$
0.1	0.8	0.1	0.1	1.0	1.0	3.0	5.0	− 4.662746	1.549648
0.5								− 3.434001	1.869631
1.0								− 2.604781	2.050189
	0.1							− 0.435967	0.482195
	0.5							− 2.355928	0.219670
	1.0							− 6.590257	0.066353
		0.1						− 4.662746	1.549648
		0.2						− 5.332671	1.080838
		0.3						− 5.735339	0.799642
			0.1					− 2.662746	1.549648
			0.2					− 3.404979	− 1.436992
			0.3					− 4.662746	− 16.690364
				1.0				− 4.662746	1.549648
				2.0				0.263420	1.967735
				3.0				0.670602	2.098724
					1.0			− 4.662746	1.549648
					3.0			− 0.670482	2.252515
					5.0			1.927269	3.352975
						− 10		− 0.260109	3.295445
						0		1.401976	1.970553
						10		2.661075	0.870973
							1	0.843098	0.905405
							3	− 1.257453	1.467243
							5	− 4.662746	1.549648

## Conclusion

The main topic of this research is to investigate the MHD and permeability effects on CNT-based nanofluids. SWCNTs and MWSNTs are suspended in blood (base fluid). Laplace transform is a very powerful mathematical tool used in various fields of physics and electrical power engineering. The Laplace transform is very important in circuit analysis, system modeling, analog signal processing, digital signal processing, process control, and radioactive decay, etc. Laplace transform technique is used to solve the non-dimensional fractional model. The exact solution for the velocity, temperature and thermal layer thickness is obtained by the above method. Zakian's algorithm is used for the simulations and inverse Laplace transform. The physical parameters such as skin fraction (drag force) and Nusselt number (heat transfer rate) are also studied. The conclusions of this study are presented below:

- The temperature of the nanofluid is higher due to the increasing estimate of the volume fraction parameter $\ddot{\varphi }$, fractional parameter $\beta$, and time $t$.
- The greater the value of the heat source or heat sink, the lower the temperature curve.
- The velocity of nanofluids in escalating function as we estimate the volume escalated fractional parameter $\ddot{\varphi }$, fractional parameters $\alpha {\text{ and }}\beta$, time $t$, and $\gamma .$
- The velocity of the nanofluid is deescalated to increase the estimate of the magnetic factor $M$ and permeability parameter $K$ due to high drag forces.
- Nanofluid temperature is higher for SWCNTs, while the reverse effects on velocity are seen.
- The thermal boundary layer increases against the fractional parameter $\beta .$
- The heat transport rate is lower for both SWCNTs and MWCNTs as a function of fraction parameters $\beta$ and $\ddot{\varphi }$ higher as a function of heat source/sink and time.
- Reinforcement occurs in the skin fraction with the growing estimate of $M,K,\alpha {\text{ and }}\gamma$ while boosting the value of $\beta ,\ddot{\varphi },Q{\text{ and }}t,$ reduces the skin fraction.

In future, we will study, what will be the effects of various fractional operators on free convective trickling over a porous medium of nanofluids with MHD and heat source/sink.

## Data Availability

All data generated or analyzed during this study are included in this article.

## List of symbols

$$\overset{\lower0.5em\hbox{$\smash{\scriptscriptstyle\frown}$}}{\tilde{\rho }}_{nf}$$: Density of nanofluid
$${\mathbf{r}}$$: Darcy’s resistance
$$\overset{\lower0.5em\hbox{$\smash{\scriptscriptstyle\frown}$}}{\tilde{\mu }}_{nf}$$: Absolute viscosity of nanofluid
$B_{0}^{2}$: Magnetic field strength
$${\mathbf{J}}$$: Current density
${\mathbf{B}}$: Total magnetic field
$$\alpha ,\beta$$: Fractional parameters
$K$: Permeability of medium
$$M$$: Magnetic parameter
$S$: Extra stress tensor
$${\rm I}$$: Unit tensor
$$T_{\infty }$$: Ambient temperature
$$\nu_{f}$$: Kinematic viscosity
$$Y$$: Space coordinates
$$T\left( {Y,\tilde{t}} \right)$$: Temperature field
LT: Laplace transforms
$$f$$: Base fluid
$$\overset{\lower0.5em\hbox{$\smash{\scriptscriptstyle\frown}$}}{\tilde{\sigma }}_{nf}$$: Electrical conductivity of nanofluid
$\left( \beta \right)_{nf}$: Thermal expansion of nanofluid
$g$: Acceleration due to gravity
$\left( {C_{p} } \right)_{nf}$: Specific heat of nanofluid
$Q^{ * }$: Coefficient of heat source/sink
$k_{nf}$: Thermal conductivity of nanofluid
$$\gamma$$: Dimensional constant
${{\varvec{\uptau}}}$: Cauchy stress tensor
$$\Pr$$: Prandtl number
$${\rm P}$$: Pressure
$U_{0}$: Amplitude of the motion
$\varphi$: Volume fraction parameter
$q_{w}$: Heat passing from surface of wall
$$\tilde{t}$$: Time
$$W\left( {Y,\tilde{t}} \right)$$: Velocity field
$$nf$$: Nanofluid
$s$: Solid nano-particles

## References

[1] Kakaç S, Pramuanjaroenkij A (2009). Review of convective heat transfer enhancement with nanofluids. Int. J. Heat Mass Transf..

[2] Khan NS, Gul T, Islam S, Khan I, Alqahtani AM, Alshomrani AS (2017). Magnetohydrodynamic nanoliquid thin film sprayed on a stretching cylinder with heat transfer. Appl. Sci..

[3] Ghosh SK, Bég OA (2008). Theoretical analysis of irradiative effects on transient free convection heat transfer past a hot vertical surface in porous media. Nonlinear Anal. Model. Control.

[4] Fetecau C, Vieru D, Azhar WA (2017). Natural convection flow of fractional nanofluids over an isothermal vertical plate with thermal radiation. Appl. Sci..

[5] Toki CJ, Tokis JN (2007). Exact solutions for the unsteady free convection flows on a porous plate with time-dependent heating. Z. Angew. Math. Mech..

[6] Hussanan A, Khan I, Shafie S (2013). An exact analysis of heat and mass transfer past a vertical plate with Newtonian heating. J. Appl. Math..

[7] Turkyilmazoglu M, Pop I (2013). Heat and mass transfer of unsteady natural convection flow of some nanofluids past a vertical infinite flat plate with radiation effect. Int. J. Heat Mass Transf..

[8] Pramanik S (2014). Casson fluid flow and heat transfer past an exponentially porous stretching surface in presence of thermal radiation. Ain Shams Eng. J..

[9] Turkyilmazoglu M (2014). Unsteady convection flow of some nanofluids past a moving vertical flat plate with heat transfer. J. Heat Transfer.

[10] Ge-JiLe H, Shah NA, Mahrous YM, Sharma P, Raju CSK, Upddhya SM (2021). Radiated magnetic flow in a suspension of ferrous nanoparticles over a cone with brownian motion and thermophoresis. Case Stud. Therm. Eng..

[11] Kavya S, Nagendramma V, Ahammad NA, Ahmad S, Raju CSK, Shah NA (2022). Magnetic-hybrid nanoparticles with stretching/shrinking cylinder in a suspension of MoS4 and copper nanoparticles. Int. Commun. Heat Mass Transfer.

[12] Kumar MD, Raju CSK, Sajjan K, El-Zahar ER, Shah NA (2022). Linear and quadratic convection on 3D flow with transpiration and hybrid nanoparticles. Int. Commun. Heat Mass Transfer.

[13] Upadhya SM, Raju SSR, Raju CSK, Shah NA, Chung JD (2022). Importance of entropy generation on Casson, Micropolar and Hybrid magneto-nanofluids in a suspension of cross diffusion. Chin. J. Phys..

[14] Raju CSK, Ahammad NA, Sajjan K, Shah NA, Yook SJ, Kumar MD (2022). Nonlinear movements of axisymmetric ternary hybrid nanofluids in a thermally radiated expanding or contracting permeable Darcy Walls with different shapes and densities: Simple linear regression. Int. Commun. Heat Mass Transfer.

[15] Khan I, Ali F, Shafie S (2012). MHD free convection flow in a porous medium with thermal diffusion and ramped wall temperature. J. Phys. Soc. Jpn..

[16] Khan A, Khan D, Khan I, Ali F, Karim F, Imran M (2018). MHD flow of sodium alginate-based casson type nanofluid passing through a porous medium with Newtonian heating. Sci. Rep..

[17] Yirga Y, Shankar B (2015). MHD flow and heat transfer of nanofluids through a porous media due to a stretching sheet with viscous dissipation and chemical reaction effects. Int. J. Comput. Methods Eng. Sci. Mech..

[18] Gaffar SA, Prasad VR, Reddy EK (2016). MHD free convection flow of Eyring-Powell fluid from vertical surface in porous media with Hall/ionslip currents and ohmic dissipation. Alex. Eng. J..

[19] Mahmoudi AH, Pop I, Shahi M, Talebi F (2013). MHD natural convection and entropy generation in a trapezoidal enclosure using Cu–water nanofluid. Comput. Fluids.

[20] Khan I, Fakhar K, Shafie S (2011). Magnetohydrodynamic free convection flow past an oscillating plate embedded in a porous medium. J. Phys. Soc. Jpn..

[21] Jha BK, Aina B, Isa S (2015). Fully developed MHD natural convection flow in a vertical annular microchannel: An exact solution. J. King Saud Univ.-Sci..

[22] Sheikholeslami M, Shehzad SA (2017). Magnetohydrodynamic nanofluid convection in a porous enclosure considering heat flux boundary condition. Int. J. Heat Mass Transf..

[23] Fetecau C, Akhtar S, Pop I, Fetecau C (2017). Unsteady general solution for MHD natural convection flow with radiative effects, heat source and shear stress on the boundary. Int. J. Numer. Meth. Heat Fluid Flow.

[24] Zeeshan A, Ellahi R, Hassan M (2014). Magnetohydrodynamic flow of waterethylene glycol based nanofluids with natural convection through a porous medium. Eur. Phys. J. Plus.

[25] Ashorynejad HR, Shahriari A (2018). MHD natural convection of hybrid nanofluid in an open wavy cavity. Results Phys..

[26] Turkyilmazoglu M (2012). Exact analytical solutions for heat and mass transfer of MHD slip flow in nanofluids. Chem. Eng. Sci..

[27] Sheikholeslami M, Gorji-Bandpy M, Vajravelu K (2015). Lattice Boltzmann simulation of magnetohydrodynamic natural convection heat transfer of Al2O3–water nanofluid in a horizontal cylindrical enclosure with an inner triangular cylinder. Int. J. Heat Mass Transf..

[28] Azhar WA, Vieru D, Fetecau C (2017). Free convection flow of some fractional nanofluids over a moving vertical plate with uniform heat flux and heat source. Phys. Fluids.

[29] Wang F, Rehman S, Bouslimi J, Khaliq H, Qureshi MI, Kamran M, Farooq A (2021). Comparative study of heat and mass transfer of generalized MHD Oldroyd-B bio-nano fluid in a permeable medium with ramped conditions. Sci. Rep..

[30] Venkata Ramudu AC, Anantha Kumar K, Sugunamma V, Sandeep N (2022). Impact of Soret and Dufour on MHD Casson fluid flow past a stretching surface with convective–diffusive conditions. J. Therm. Anal. Calorim..

[31] Farooq A, Rehman S, Alharbi AN, Kamran M, Botmart T, Khan I (2022). Closed-form solution of oscillating Maxwell nano-fluid with heat and mass transfer. Sci. Rep..

[32] Tang R, Rehman S, Farooq A, Kamran M, Qureshi MI, Fahad A, Liu JB (2021). A comparative study of natural convection flow of fractional maxwell fluid with uniform heat flux and radiation. Complexity.

[33] Kumar KA, Sugunamma V, Sandeep N, Mustafa M (2019). Simultaneous solutions for first order and second order slips on micropolar fluid flow across a convective surface in the presence of Lorentz force and variable heat source/sink. Sci. Rep..

[34] Kumar KA, Reddy JR, Sugunamma V, Sandeep N (2018). Magnetohydrodynamic Cattaneo-Christov flow past a cone and a wedge with variable heat source/sink. Alex. Eng. J..

[35] Kumar KA, Sugunamma V, Sandeep N, Reddy J (2019). R, Numerical examination of MHD nonlinear radiative slip motion of non-newtonian fluid across a stretching sheet in the presence of a porous medium. Heat Transfer Res..

[36] Anantha Kumar K, Sugunamma V, Sandeep N (2020). Influence of viscous dissipation on MHD flow of micropolar fluid over a slendering stretching surface with modified heat flux model. J. Therm. Anal. Calorim..

[37] Anantha Kumar K, Sugunamma V, Sandeep N (2020). Effect of thermal radiation on MHD Casson fluid flow over an exponentially stretching curved sheet. J. Therm. Anal. Calorim..

[38] Kumar KA, Reddy JR, Sugunamma V, Sandeep N (2019). MHD flow of chemically reacting Williamson fluid over a curved/flat surface with variable heat source/sink. Int. J. Fluid Mech. Res..

[39] Kumar A, Sugunamma V, Sandeep N (2018). Impact of non-linear radiation on MHD non-aligned stagnation point flow of micropolar fluid over a convective surface. J. Non-Equilib. Thermodyn..

[40] Anantha-Kumar K, Sugunamma V, Sandeep N (2019). Physical aspects on unsteady MHD-free convective stagnation point flow of micropolar fluid over a stretching surface. Heat Transfer Asian Res..

[41] Zakian V (1969). Numerical inversion of Laplace transform. Electron. Lett..

[42] Fuzhang W, Muhammad IA, Mohsin A, El-Shafay AS, Nadeem A, Rifaqat A (2022). Inspections of unsteady micropolar nanofluid model over exponentially stretching curved surface with chemical reaction. Waves Random Complex Media.

[43] Wang F, Sohail AK, Soumaya G, Essam R, El-Zahar M, Ijaz K, Sami U, Khan MY, Yong-Min L (2022). Entropy optimized flow of Darcy-Forchheimer viscous fluid with cubic autocatalysis chemical reactions. Int. J. Hydrogen Energy.

[44] Wang F, Rangaswamy NK, Ballajja CP, Umair K, Aurang Z, Abdel-Haleem A-A, Ibrahim SY, Mohammed SA, Ahmed MG (2022). Aspects of uniform horizontal magnetic field and nanoparticle aggregation in the flow of nanofluid with melting heat transfer. Nanomaterials.

[45] Wang F, Muhammad IA, Muhammad Z, Azhar I, Hijaz A, Alsulami MD (2021). Unsteady thermal transport flow of Casson nanofluids with generalized Mittag-Leffler kernel of Prabhakar's type. J. Mater. Res. Technol..

[46] Wang F, Enran H, Samir AS, Mostafa MAK (2022). Numerical investigation of the nonlinear fractional Ostrovsky equation. Fractals.

